# Triple Trouble: Hepatitis B-related Cirrhosis With Hodgkin Lymphoma-Associated Paraneoplastic Pseudoachalasia

**DOI:** 10.7759/cureus.100742

**Published:** 2026-01-04

**Authors:** Venkatesh Vaithiyam, Ravi Teja Reddy, Ashok Dalal, Surbhi Goyal, Sanjeev Sachdeva

**Affiliations:** 1 Gastroenterology, Govind Ballabh Pant Institute of Postgraduate Medical Education and Research, New Delhi, IND; 2 Pathology, Govind Ballabh Pant Institute of Postgraduate Medical Education and Research, New Delhi, IND

**Keywords:** achalasia cardia, dysphagia, hepatitis b, hodgkin’s lymphoma, pseudoachalasia

## Abstract

Pseudoachalasia is a rare motility disorder that occurs secondary to various disorders, including malignancies. Clinical, endoscopic, and manometric features of pseudoachalasia mimic primary achalasia. Paraneoplastic pseudoachalasia is an uncommon form of pseudoachalasia associated with malignancies, such as small cell lung carcinoma, neuroendocrine tumors, acute leukemia, and lymphomas. Symptoms of paraneoplastic pseudoachalasia can occur early, together, or delayed compared to those of primary malignancy. The pathophysiology of paraneoplastic achalasia involves autoantibody-induced destruction of the myenteric plexus of the esophagus. We report a case of an 18-year-old male who presented with dysphagia, anorexia, weight loss, and ascites, which were suggestive of achalasia. Further investigations revealed hepatitis B-related cirrhosis with portal hypertension and enlarged para-aortic and aorto-caval lymph nodes. Endoscopic ultrasound-guided fine-needle biopsy of the lymph nodes confirmed the diagnosis of Hodgkin's lymphoma. High-resolution manometry revealed type II achalasia cardia, which is likely a paraneoplastic manifestation of Hodgkin's lymphoma. The patient underwent endoscopic pneumatic dilation for symptom relief and was referred for further management of Hodgkin's lymphoma. This case highlights the importance of considering paraneoplastic pseudoachalasia in patients presenting with achalasia-like symptoms, especially in the presence of significant weight loss and anorexia.

## Introduction

Idiopathic (primary) achalasia is a common primary motility disorder of the esophagus caused by the loss of inhibitory neurons in the myenteric plexus [1]. It is associated with aperistalsis of the esophagus and impaired relaxation of the lower esophageal sphincter (LES). The clinical features of achalasia include dysphagia, regurgitation, chest pain, and weight loss. The pathogenesis of achalasia is not yet fully understood. Pseudoachalasia, also known as secondary achalasia, is a clinical condition mimicking achalasia that occurs secondary to a wide range of disorders, such as metastatic solid organ malignancies, lymphoproliferative disorders, and post-surgical conditions [2]. Paraneoplastic achalasia is a type of pseudoachalasia caused by a paraneoplastic autoimmune response in which tumor-associated antineuronal antibodies cause immune-mediated destruction of the esophageal myenteric plexus without direct tumor infiltration or mechanical obstruction. Paraneoplastic pseudoachalasia has been reported in lung and hematological malignancies, such as acute leukemia and lymphoma. The distinction between secondary and primary achalasia is important, as misdiagnosis may lead to the progression of the underlying malignancy and poor survival rates. Primary and secondary achalasia usually have similar clinical, endoscopic, and manometric findings [3]. However, patients with pseudoachalasia are older than those with primary achalasia and often present with a short history of disproportionate weight loss. Herein, we describe the case of a young man who presented with symptoms suggestive of achalasia, chronic liver disease, and Hodgkin’s lymphoma.

## Case presentation

**Figure 1:**
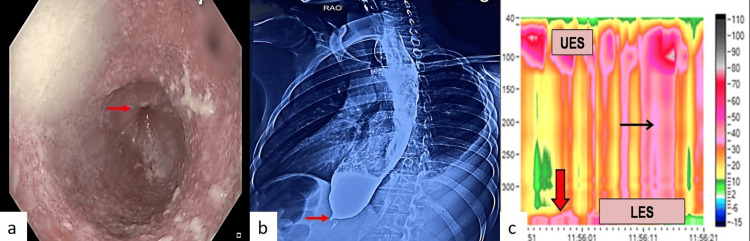
a) Upper gastrointestinal endoscopy showing dilated esophagus with liquid food residue and tight lower esophagus sphincter (red arrow). b) Barium swallow radiograph showing dilated esophagus and narrowing of lower esophagus sphincter (red arrow). c) High-resolution manometry shows increased integrated relaxing pressure (red arrow) and pan-esophageal pressurization (black arrow).

An 18-year-old male with no addiction or prior comorbidities presented with an 11-month history of dysphagia, anorexia, weight loss, and regurgitation for six months. The dysphagia was progressive, initially affecting liquids and later solids, and was associated with mild retrosternal discomfort. The patient also complained of anorexia and a weight loss of approximately 20 kg. He also complained of abdominal distention for the past two months, which had gradually progressed. Physical examination revealed pallor, severe cachexia (body mass index, 13.2 kg/m^2^), and ascites. Blood investigations showed pancytopenia and hypoalbuminemia. Ascitic fluid analysis revealed a high serum ascitic albumin gradient with low protein content, and ultrasonography confirmed cirrhosis with portal hypertension. Evaluation for the etiology of cirrhosis demonstrated hepatitis B infection, with negative hepatitis C and antinuclear antibody tests. Hepatitis B serology revealed positive HbsAg, negative HBeAg, positive anti-HBe, and an HBV DNA level of 28,000 IU/mL, suggesting hepatitis B as the underlying cause of cirrhosis. Upper GI endoscopy revealed a dilated esophagus with liquid food residue and resistance at the LES, but no varices (Figure 1a). Barium swallow showed features characteristic of achalasia cardia, including a dilated esophagus with a hold-up of contrast and smooth narrowing at the GE junction (Figure 1b). High-resolution manometry revealed features of type II achalasia cardia, according to the Chicago classification (Figure 1c).

**Figure 2:**
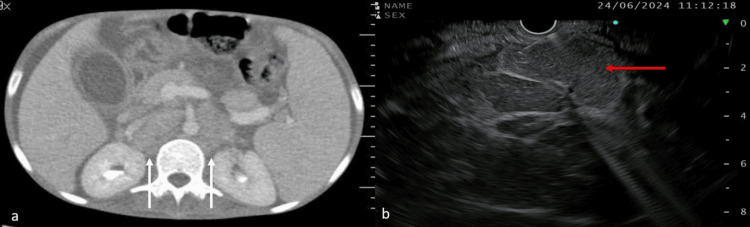
a) Contrast-enhanced computed tomography image showed multiple aorto-caval and paraaortic lymph nodes (white arrow); note is made for ascites. (b) Endoscopic ultrasound image shows an enlarged aorto-caval lymph node (red arrow).

Because of the significant weight loss and anorexia, we suspected pseudoachalasia in our patient. Computed tomography of the chest and abdomen was performed, which confirmed the presence of cirrhosis and enlarged para-aortic and aorto-caval lymph nodes (Figure 2a). Endoscopic ultrasound showed multiple enlarged para-aortic and aorto-caval lymph nodes, and fine-needle biopsy of the lymph node was performed (Figure 2b).

**Figure 3:**
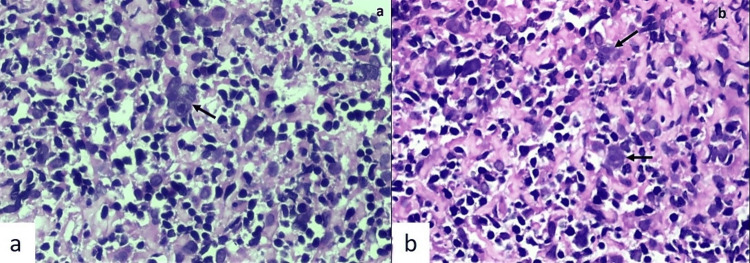
a) Core biopsy showed atypical large multinucleate Reed-Sternberg (RS) like cells (black arrow) having amphophilic cytoplasm, round nucleus with a prominent cherry red macronucleolus scattered in a background of lymphocytes and plasma cells (H&E ×400). b) Few mononuclear variants of RS cells highlighted by black arrow (H&E ×400).

Histopathological examination revealed atypical, large, mononuclear Reed-Sternberg (RS)-like cells with amphophilic cytoplasm and round nuclei with a prominent cherry-red macronucleolus, scattered in a background of lymphocytes, plasma cells, and occasional eosinophils. A few multinucleate and occasional binucleate RS cells were also observed (Figures 3a-3b). 

**Figure 4:**
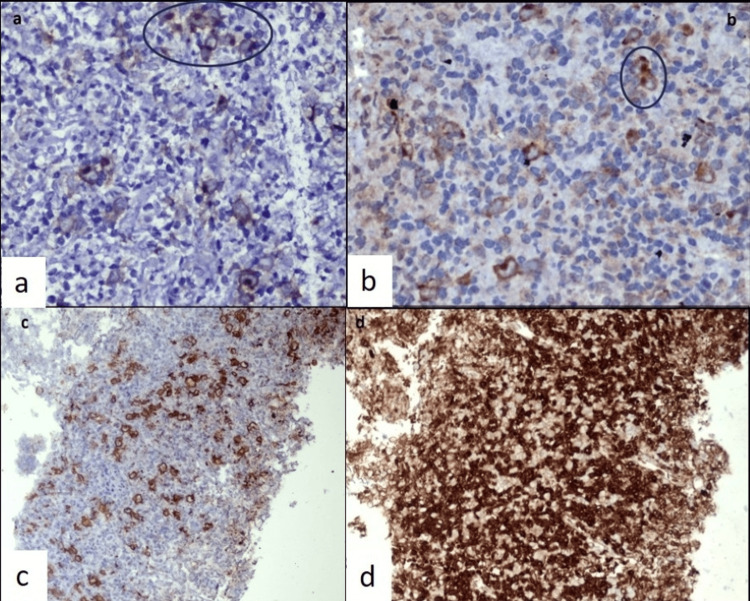
On immunohistochemistry, the Reed-Sternberg cells showed a) characteristic membranous and Golgi pattern of immunoexpression for CD 30, b) cytoplasmic expression with Golgi accentuation for latent membrane protein-1 (LMP-1) of Epstein-Barr virus (EBV), c) variable CD20 expression with incomplete membranous staining and background lymphocytes were predominantly negative, and d) background lymphocytes showed predominant diffuse CD3 expression suggestive of classical Hodgkin lymphoma.

These cells showed characteristic membranous and Golgi patterns of immunoexpression for CD30, cytoplasmic expression with Golgi accentuation for latent membrane protein-1 (LMP-1) of the Epstein-Barr virus (EBV), variable CD20 expression with incomplete membranous staining, and were negative for CD15 and CD3. Background lymphocytes showed predominant CD3 expression and were negative for CD20, confirming the diagnosis of classical Hodgkin lymphoma (Figures 4a-4d).

He was started on tenofovir for hepatitis B infection, along with supportive management for complications of cirrhosis. In view of the poor performance status, we performed endoscopic pneumatic dilation for symptom relief. With the above management, his dysphagia improved, and he had significant weight gain. He was referred to the medical oncology unit for further management of Hodgkin’s lymphoma.

## Discussion

The term “achalasia” is derived from the Greek word “khalasis,” whose meaning is “relaxation.” Achalasia is a common motor disorder of the esophagus characterized by incomplete LES relaxation and impaired peristalsis without any evidence of mechanical obstruction. The pathogenesis involves degeneration of ganglion cells in the myenteric plexus of the esophageal body and LES. The etiology of primary achalasia is unknown; however, genetic, viral, autoimmune, and primary neurodegenerative processes are hypothesized to be etiological factors. The incidence of achalasia is approximately 1.6 cases per 100,000 individuals. The incidence increases with age, peaking between 40 and 60 years, and is equally distributed between both sexes. Pseudoachalasia refers to a wide range of disorders that clinically, manometrically, and radiographically resemble primary achalasia. The incidence of pseudoachalasia is approximately 2-4% among patients with manometric findings suggestive of achalasia [4]. The first case report of pseudoachalasia as a manifestation of malignancy was described by Howarth in 1919 [5]. Primary or secondary malignancy is the most common cause of pseudoachalasia in 70% of affected patients, and it is higher in primary malignancies (54-70% of cases) than in secondary malignancies (6% of cases) [4]. The most common malignancy associated with pseudoachalasia is carcinoma of the esophagus or gastric cardia [2,3]. In a case series by Katzka et al., esophageal adenocarcinoma was the most common malignancy among 17 cases of secondary achalasia, followed by breast and non-small cell lung carcinomas [6]. Submucosal invasion with secondary destruction of the myenteric plexus and loss of ganglion cells is the proposed mechanism underlying malignancy-associated pseudoachalasia [7].

Malignancy-associated pseudoachalasia can occur due to paraneoplastic syndromes. The incidence of paraneoplastic achalasia is 0.4% among achalasia patients and 2.5% among secondary achalasia patients [8,9]. These paraneoplastic syndromes can present with isolated involvement of the esophagus or as part of generalized gastrointestinal motility disorders [10]. The pathophysiology of paraneoplastic achalasia involves autoantibody-induced destruction of the myenteric plexus of the esophagus. Type 1 antineuronal nuclear autoantibodies (anti-Hu antibodies) are the most common; anti-Yo and N-type Ca2+ channel antineuronal antibodies have also been implicated [2]. Paraneoplastic enteric neuropathy is a progressive disease that is reversible during the early stages. In the late stages, chronic inflammation results in the degeneration of neuronal ganglia cells, subsequent apoptosis, and loss of ganglion cells. The common malignancy associated with paraneoplastic pseudoachalasia is small cell carcinoma of the lung, neuroendocrine tumors, acute leukemia, Hodgkin’s and non-Hodgkin’s lymphoma [11-13]. Hodgkin's lymphoma can be associated with multiple paraneoplastic neurological syndromes, such as cerebellar degeneration, acute and chronic inflammatory demyelinating polyneuropathy, motor neuron diseases, myasthenia gravis, and achalasia cardia. Our patient had hepatitis B-related cirrhosis, but hepatitis B virus infection is usually associated with non-Hodgkin lymphoma [14]. EBV is commonly associated with Hodgkin’s lymphoma, whereas the role of hepatitis B in etiopathogenesis is unclear [15]. 

It is important to differentiate between primary and pseudoachalasia, as their prognoses vary significantly; however, such a distinction is difficult. In paraneoplastic achalasia, the onset of GI dysmotility and its symptoms can precede the diagnosis of primary malignancy [10,12]. In our patient, achalasia-like symptoms preceded the diagnosis of Hodgkin’s lymphoma by 11 months. Older age and shorter symptom duration indicate the presence of pseudoachalasia. In a case series by Woodfield et al. of 39 patients, 10 with secondary achalasia, all patients with secondary achalasia had symptoms lasting less than four months, whereas >95% of patients with primary achalasia had dysphagia for > 12 months [16]. Another study by Tracey et al. also found a significant difference in the duration of dysphagia among pseudoachalasia (9.6 ± 8.6 months) and primary achalasia cases (54.3 ± 44.2 months) [17]. Our patient was young, with a short duration of symptoms (11 months). Disproportionate weight loss and severe cachexia are other important pointers towards pseudoachalasia. In a study by Rozman et al., weight loss was reported more in secondary achalasia than primary achalasia (88.2% versus 57.3 %, respectively) [18]. In secondary achalasia, weight loss is greater despite a shorter duration of illness [19]. Our patient had anorexia and a weight loss of approximately 20 kg over 11 months. Higher Eckardt symptoms have also been associated with malignant pseudoachalasia [2].

The treatment of paraneoplastic achalasia includes management of the underlying malignancy and therapies aimed at lowering the LES pressure. Management of primary neoplasia alone usually does not result in significant improvement in achalasia, as autoantibody-mediated destruction of the myenteric plexus occurs. There is no preventive or definitive curative treatment for myenteric esophageal plexus degeneration. In a recent case series by Abbasi et al., small-cell lung carcinoma was the most common underlying neoplastic lesion, followed by intestinal malignancies and neuroendocrine tumors as causes of paraneoplastic achalasia. In this cohort, antineuronal antibodies were positive in 76.5% of patients, and all cases were successfully treated with peroral endoscopic myotomy (POEM), suggesting both a paraneoplastic immunological mechanism and the efficacy of POEM as a therapeutic option in this setting [20]. Our patient had a poor performance status and underwent pneumatic dilatation with a plan to perform an oral endoscopic myotomy once his general condition improved, followed by chemotherapy for Hodgkin’s lymphoma.

## Conclusions

Paraneoplastic achalasia is an uncommon form of achalasia and an important cause of pseudoachalasia. Although less frequent than primary achalasia, it is not rare and should be considered in patients presenting with a short duration of symptoms, disproportionate weight loss, and severe cachexia, as these features suggest secondary achalasia. Importantly, the symptoms of paraneoplastic achalasia may precede the clinical detection of the underlying malignancy, underscoring the need for a high index of suspicion and thorough evaluation in such cases.
